# Causal Inference with Case-Only Studies in Injury Epidemiology Research

**DOI:** 10.1007/s40471-022-00306-8

**Published:** 2022-09-29

**Authors:** Andrew G. Rundle, Michael D. M. Bader, Charles C. Branas, Gina S. Lovasi, Stephen J. Mooney, Christopher N. Morrison, Kathryn M. Neckerman

**Affiliations:** 1 Department of Epidemiology, Mailman School of Public Health, Columbia University, 722 West 168th Street, Room 727, New York, NY 10032, USA; 2 Department of Sociology, Johns Hopkins University, Baltimore, MD, USA; 3 Department of Epidemiology and Biostatistics, Drexel University, Philadelphia, PA, USA; 4 Department of Epidemiology, University of Washington, Seattle, WA, USA

**Keywords:** Study design, Case-only design, Injury research, Pedestrian injury, Etiologic heterogeneity, Effect modification

## Abstract

**Purpose of Review:**

We review the application and limitations of two implementations of the “case-only design” in injury epidemiology with example analyses of Fatality Analysis Reporting System data.

**Recent Findings:**

The term “case-only design” covers a variety of epidemiologic designs; here, two implementations of the design are reviewed: (1) studies to uncover etiological heterogeneity and (2) studies to measure exposure effect modification. These two designs produce results that require different interpretations and rely upon different assumptions. The key assumption of case-only designs for exposure effect modification, the more commonly used of the two designs, does not commonly hold for injuries and so results from studies using this design cannot be interpreted. Case-only designs to identify etiological heterogeneity in injury risk are interpretable but only when the case-series is conceptualized as arising from an underlying cohort.

**Summary:**

The results of studies using case-only designs are commonly misinterpreted in the injury literature.

## Introduction

Retrospective designs that compare cases to comparison groups are well suited to injury epidemiology for two important reasons: injury outcomes are rare at the population level, meaning prospective study is often infeasible; and the latent period between putative causes and injury outcomes is often very short, so it is justifiable to assess exposure and outcome status at the same time. The term “case-only design” has been applied to a wide variety of retrospective study designs, two of which, the case-only design to investigate etiological heterogeneity [1] and the case-only design to measure exposure effect modification [2, 3], have been frequently used in injury epidemiology (for instance, see [4–11]). These two designs, also called case-series, case-case or case^2^ studies, share a common design strategy, in which a case-series is stratified into two or more case groups, which are then compared in regard to some putative predictor variable [1, 2, 12]. The variable used to stratify the case-series into sub-groups is the dependent variable in the case-only analyses [1, 2]. For instance, in a case-series of automobile crashes, data on blood alcohol levels of drivers can be used to divide crashes into those with drivers under the influence of alcohol and those with drivers who were not [13]. Logistic regression analyses are then performed to determine whether predictor variables (e.g., age of driver) are associated with one or other of the two automobile crash sub-types. These two case-only designs are attractive for injury epidemiology because of the difficulties in selecting controls and gathering data on exposures of interest for injury, which are often of a sensitive nature, such as alcohol and drug use, risk-taking behaviors, mental health issues, and adverse life events [14, 15].

Because the phrase “case-only design” has been used in the literature to refer to a variety of different designs, the reader is cautioned to distinguish these two designs from other designs sometimes referred to as “case-only designs.” Other designs sometimes referred to as “case-only” are case-series that are observed at two time points for exposure status (AKA, case-crossover, self-controlled case series, self-controlled risk interval) or a case-series study in which the proportion of exposed cases is so large, it obviously differs from the general population (e.g., chimney sweeping among scrotal cancer patients) [16]. Furthermore the two designs reviewed here are both referred to in the literature as “case-only designs” and employ similar regression-based analytical approaches, but they generate statistical parameters calling for different interpretations [1, 2, 17]. Furthermore, the odds ratios (ORs) generated from the case-only regression analyses used in these two designs carry very different meanings than the OR parameters estimated by the case-control or cohort analyses [1, 2, 18]. Here, we review the case-only design to investigate etiological heterogeneity [1] and the case-only design to measure exposure effect modification [2, 3]. We describe the value of each design and then describe how each may be used in injury epidemiology, providing example analyses of Fatality Analysis Reporting System (FARS) data on pedestrian fatalities.

## How to Interpret Results From These Two Case-Only Designs

To clarify interpretations of case-only parameters, it is worth reviewing why case-control studies offer estimates of causal effects. Case-control studies are valid and interpretable because they can be interpreted as stratified selection from an underlying cohort, and so have the potential to produce unbiased estimates of causal effects of risk factors on outcomes. If done correctly, with samples of cases and controls properly chosen to represent the target population that the study is intended to reflect, case-control studies can be seen as efficient versions of larger, and often time- and resource-prohibitive, cohort studies [3, 19, 20]. It is understood that odds ratios from case-control studies are valid estimates of risk ratios or rate ratios that would have otherwise been estimated from a cohort study that generated the same case-series as analyzed in the case-control study [3]. Estimates identified in case-control studies generalize to the population when the case and control samples represent the exposure history of cases and controls in a broader target population of interest.

**Fig. 1:**
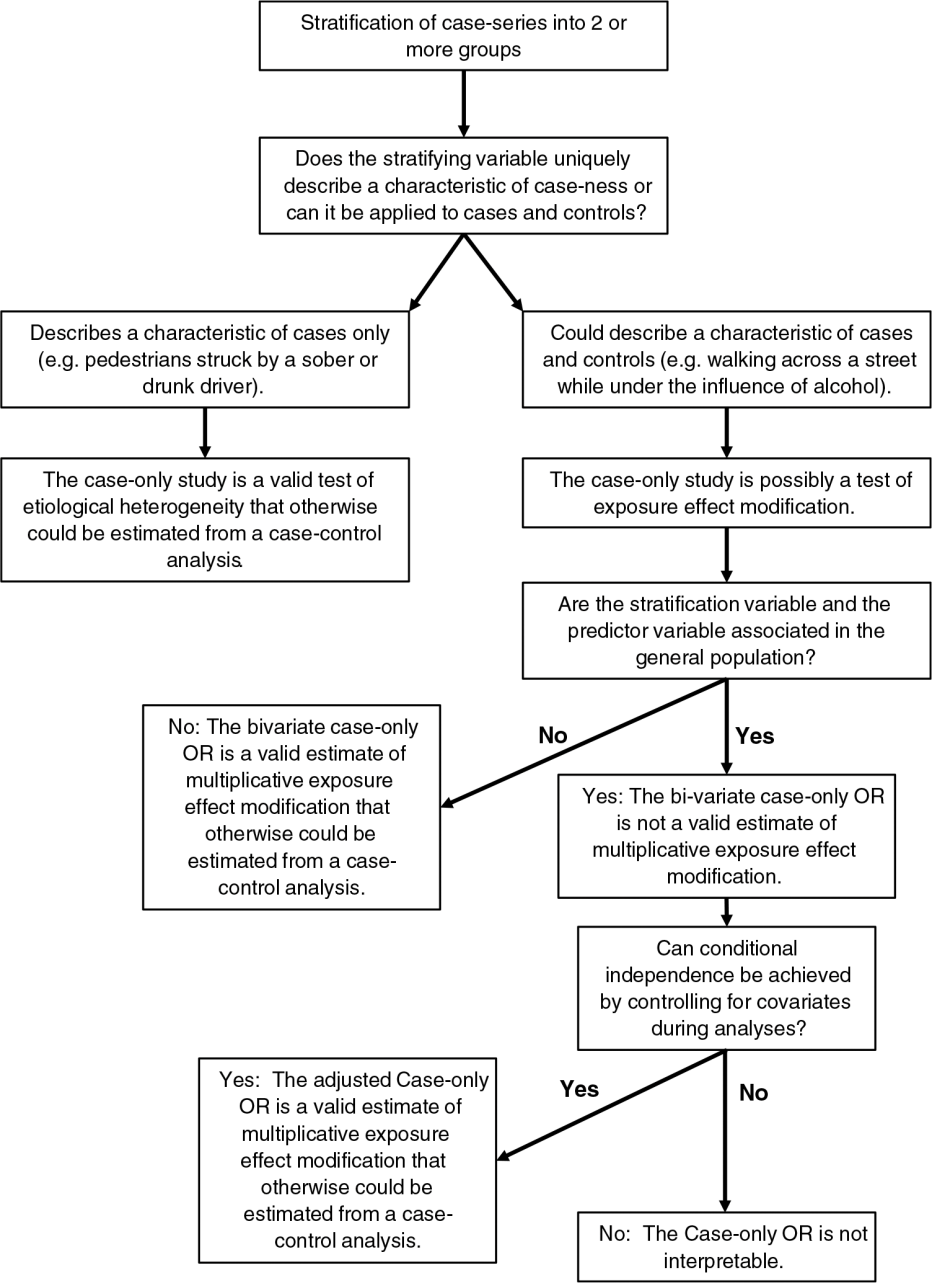
Flow chart for conducting and interpreting a case-only analysis

To be interpretable, the two case-only designs reviewed here must be understood to utilize the same case-series that otherwise would have been utilized in a case-control study, or if the case-control study were nested in an extant cohort, the case-series that would have been generated from the cohort [1–3, 18]. However, the odds ratio estimated by these case-only designs does not estimate the causal effect of a risk factor on an outcome: to accomplish this, an epidemiologically sound control series is required. The estimand in these two case-only design depends on the type of variable used to stratify or group the case-series. In case-only studies of *etiological heterogeneity*, the stratifying variable describes some inherent characteristic of case-ness that does not have a logical or comparable value for non-cases [1]. In the case-only study of *exposure effect modification*, the stratifying variable can be used to describe both cases and non-cases and could be analyzed as a risk factor for the overall outcome in a full cohort or case-control study [2, 3]. Figure 1 provides a flow chart for conducting and interpreting case-only designs. Discussions of the results of case-only analyses in injury epidemiology rarely state the hypothesis being tested by the design and rarely relate the estimated case-only OR to the OR that would have been estimated in case-control analyses of the case-series under investigation. As such, we argue that the results of these two case-only designs are commonly misinterpreted in injury epidemiology.

## Etiological Heterogeneity

The case-only study of etiological heterogeneity tests whether a risk factor has a different causal effect for one case-subtype compared to another case-subtype [1]. It can be used to identify potential mechanisms that may explain how different forms of an outcome come about [1]. It cannot, however, determine population-level risk of first experiencing or contracting those outcomes [1, 12].

Begg and Zhang originally described this design in their study of smoking’s influence on whether a patient had one sub-type of bladder cancer verses another subtype [1, 21]. In this study, cases were classified into two groups based on the presence or absence of a mutation in the p53 gene in the tumor tissue, a classification that had no meaning for controls, as by definition controls have no bladder tumor tissue. Begg and Zhang showed that the odds ratio (OR) for smoking on p53+ status from the case-only design, θ_Case-Only_, was equivalent to the ratio of two case-control ORs for smoking estimated from a full case-control study: the OR when p53+ cancer cases were compared to controls, θ_1_, and the OR when p53- cancer cases were compared to controls, θ_2_.[1] That is, θ_Case-Only_ = θ1/θ2.

A logistic regression model predicting tumor p53+ status based *on smoking among cases only is*:

(1) $$\log[\text{P}(\text{Y})/(1-\text{P}(\text{Y}))]=\text{α}+{\text{βX}}_{\text{i}}$$

where *Y* represents a case with the p53 mutation and *X*_*i*_ represents whether subject *i* was a current smoker and e^β^ = θ_Case-Only_ = θ_1_/θ_2_, which can equivalently be estimated from the case-only analyses or case-control analyses of the same case series. The exponentiated coefficient from the case-only model is interpreted as describing the extent to which an exposure differs in its effect on one subtype of cases compared to another, a phenomenon described as etiological heterogeneity [1]. The case-only odds ratio, *e*^*β*^, only reflects the extent to which there is a *difference* in the effect of an exposure between the two subtypes of cases. It does not provide information about the effect of smoking on the risk of getting bladder cancer. An *e*^*β*^*case-only* = 2 could indicate that θ_1_ = 2 and θ_2_ =1 or that θ_1_ = 3 and θ_2_ =1.5 or even θ_1_ = 1 and θ_2_ =0.5; thus, it informs us on the ratio of θ_2_ to θ_1_ but not the values of θ_2_ and θ_1_ [1].

The rationale for controlling for covariates in a case-only design of etiologic heterogeneity is similar to that in a traditional case-control or cohort study; there is a class of variables that if uncontrolled for will cause bias in the estimate of the case-only OR for the exposure of interest. Such variables are similar to traditional confounders in that they are associated with the exposure variable of interest, but to “confound” a case-only study of etiologic heterogeneity and create an omitted variable bias, they must also show etiologic heterogeneity for the sub-grouped outcomes in question. If adjustment for a potential confounder causes a similar attenuation for both θ_1_ and θ_2_, such as with θ_1_ being attenuated from 4 to 3 after adjustment and θ_2_ being attenuated from 2 to 1.5, their ratio may be unchanged (with θ_1_/θ_2_ remaining 2 both before and after adjustment in this case).

### Exposure Effect Modification

The case-only study of exposure effect modification tests whether one exposure modifies the effect of another exposure (or an intrinsic characteristic of the study participant such as age) [2, 3, 18]. This design measures the extent of multiplicative interaction between two exposures that would otherwise be estimated using a case-control or cohort study, under the assumption that there is no association between the two exposures in the underlying source population (the independence assumption) [2, 3, 18]. If the independence assumption does not hold, the univariate OR from a case-only analysis of exposure effect modification is not interpretable. An advantage of the case-only design is that, if the independence assumption holds, it provides a more statistically efficient estimate of the multiplicative interaction term than would otherwise be generated from case-control analyses of the same case series [2]. This design was first described in the context of gene-by-environment interactions, where the independence assumption was thought to commonly hold [2, 3, 18]. Examples are case-only studies of the interaction between the N-acetyltransferase 2 (NAT-2) genetic polymorphisms and cigarette smoking on bladder cancer risk [17]. Its utility has expanded to include interactions between other pairs of risk factor variables including gene-by-gene and environment-by-environment. Unlike case-only studies of etiological heterogeneity, case-only designs for estimating exposure effect modification use variables that measure exposures and/or characteristics observable in both cases and controls [3, 18].

In the underlying population from which cases arise, two risk factors (e.g., the NAT-2 genetic polymorphism and cigarette smoking) may interact to affect the odds of an outcome (e.g., bladder cancer). If so, the population may be modeled by Eq. (2):

(2) $$\log[\text{P}(\text{Y})/(1-\text{P}(\text{Y}))]=\text{α}+{\text{β}}_{1}\text{X}+{\text{β}}_{2}\text{Z}+{\text{β}}_{3}{\text{X}}^{*}\text{Z}$$

A cohort or case-control study could be devised to estimate such a model, since X and Z can be observed in both cases and controls or in an entire cohort. For example, X may represent a gene (e.g., NAT-2) that modifies the effect of an environmental or behavioral risk factor, Z (e.g., smoking) on disease risk (e.g., bladder cancer) [17].

Using a case-only design, we could estimate the effect modification of X on Z based on predicting the presence of X among cases based on Z under the assumption that X and Z are conditionally uncorrelated among (unobserved) controls. From the example of bladder cancer risk, the OR for the association between NAT-2 genetic polymorphism status (X) and cigarette smoking status (Z) among cases was calculated [17]. In the Eq. (3) below, implemented among cases only, γ_1_ is equivalent to β_3_ in Eq. (2):

(3) $$\log[\text{P}(\text{X})/(1-\text{P}(\text{X}))]=\gamma_{0}+\gamma_{1}\text{Z}$$

The rationale for controlling for covariates in a case-only study of exposure effect modification is very different from the rationale for controlling for covariates in a cohort or case-control study [18]. In circumstances where X and Z are associated in the underlying population, covariate control can be used in case-only analyses to establish conditional independence between X and Z, so that a non-biased estimate of the magnitude of effect modification between X and Z can be generated [18]. In practical terms, this means conceptualizing why X and Z are associated in the underlying population and identifying a variable(s), M, such that X and Z are independent, conditional on M [18]. This variable(s) M is then included in the case-only analysis as a covariate, with case-only logistic regression model taking the form of log[P(X)/(1−P(X))] = γ_0_ + γ_1_Z + γ_2_M; however, the OR for M is not of interest itself and is not interpretable.

A multivariate case-only analysis that includes multiple covariates, e.g., Z_1_, Z_2_, …, Z_k_, each conceptualized as an exposure, would generate a series of corresponding ORs measuring the effect modification of each Z_k_ on X. A case-only logistic regression model of the form log[P(X)/(1−P(X))] = γ_0_ + γ_1_Z_1_ + γ_2_Z_2_, where Z_2_ is another exposure, estimates the X*Z_1_ and X*Z_2_ interaction terms from the model log[P(Y)/(1−P(Y))] = α + β_1_X + β_2_Z_1_ + β_3_Z_2_ + β_4_X*Z_1_ + β_5_X*Z_2_ fit in a case-control study, where X and Z_1_, and X and Z_2_, are assumed to be unassociated in the general population. Practically, however, interpreting multiple effect modifiers of X quickly becomes unwieldy.

### Implications for Case-Series Analyses in Injury Epidemiology

When these two case-only designs are implemented in injury epidemiology, we argue, the analyses are commonly misinterpreted, most notably because researchers overlook the independence assumption required for the case-only studies of effect modification. Considering alcohol consumption as a risk factor for pedestrian fatality, a case-only study with the driver’s alcohol status as the stratifying variable tests a completely different type of hypothesis than a case-only study with pedestrian alcohol consumption as the stratifying variable [22–28]. In the context of these two options for stratifying a case-series on alcohol involvement, Supplemental Fig. 1 illustrates the relationship between case-only and case-control analyses for etiologic heterogeneity and Supplemental Fig. 2 illustrates the relationship between case-only and case-control analyses for multiplicative interaction. The rest of this section illustrates the application of these two case-only designs using analyses of 2017 and 2018 FARS data. Table 1 shows the results of two case-only analyses of the association between pedestrian age and two groups of pedestrian fatalities, one stratifying based on the driver’s alcohol status and the second stratifying on the pedestrian’s alcohol status.

In the first analysis, the stratifying variable is whether the *driver* involved in the accident was identified as a “drinking driver” (based either on police reports or a positive alcohol test) [29]. Driver alcohol-involvement can be used to stratify the pedestrian fatality case-series, but cannot logically be used to stratify or describe non-cases, i.e., individuals in the underlying case-control study that were not killed by an automobile. Thus, this case-only analysis is a test of *etiological heterogeneity,* that is, whether the age of the pedestrian is differentially associated with being killed by a drunk versus sober driver. Alternatively, if this case-series was analyzed within a case-control design, two sets of OR for the effect of age group on fatality risk would be calculated. The first would be calculated comparing the age distribution of pedestrians killed by drunk drivers to the age distribution of controls, and the second would be calculated comparing the age distribution of pedestrians killed by sober drivers to the age distribution of controls. The ratio of these two sets of OR for the age groups would equal the OR generated for age from case-only analyses of the case-series alone (see Supplemental Fig. 1).

The results from the first case-only analysis suggest that the effect of age on risk of pedestrian fatality is similar for crashes involving a drunk driver compared to crashes involving sober drivers, up until the age of 60+ years. These analyses suggest that compared to those age 16 to 20 years old, individuals age 60 years or older are less likely to be killed in a crash involving a drunk driver than a crash involving a sober driver. However, these analyses do not provide evidence that this older age group is at lower risk for pedestrian fatality overall. Further adjustment for pedestrian sex and race does not materially alter the ORs for age groups, because, after adjustment for age and each other, sex and race do not show substantial etiologic heterogeneity for the two pedestrian fatality sub-groups.

In the second analysis, the case-series could be conceptualized as part of a case-control study in which the alcohol status of control pedestrians is assessed [14]. Thus, this case-only analysis is a test of *exposure effect modification*. Matched controls could be enrolled, with data collected on whether they were walking outdoors and had recently consumed alcohol at the time when the case was killed [14]. These controls would allow estimation of the prevalence of individuals walking while under the influence of alcohol in the population from which the cases series arose, allowing a valid test of the association between walking while under the influence of alcohol and pedestrian fatality risk [3, 14, 20]. This case-control study could also assess multiplicative interactions between other exposures or study participant characteristics, in our example the age and alcohol consumption status of the pedestrian. The interaction effect would express the extent to which the effect of consuming alcohol and walking on the odds of being fatally struck by a car depends on the pedestrian’s age.

When the case-series is analyzed using a case-only approach, the OR estimates the extent of multiplicative exposure effect modification between the pedestrian’s alcohol consumption status and the age of the pedestrian. The estimated case-only univariate odds ratios from the FARS data in Table 1 suggest that, in a full case-control analysis, all of the age group × pedestrian alcohol status interaction terms, except for the 60+ age group, would be significantly different from 1. That is, the effect of walking after consuming alcohol on the odds of being fatally struck by a car depends on the age of the pedestrian. Critically, this interpretation of the analysis depends on the assumption that alcohol consumption and pedestrian age are not associated in the general population [2, 3]. However, survey data on alcohol consumption across age groups suggests that this independence assumption does not hold [30]. Thus, it is unlikely that the univariate OR generated in the case-only analyses of the FARS can be interpreted as reflecting valid estimates of exposure effect modification.

Valid interpretation of results from case-only studies of exposure effect modification requires careful attention to this independence assumption—the assumption that the two exposures of interest are unassociated in the source population [2, 3, 18]. The original formulation of case-only studies of exposure effect modification focused on gene-environment interactions, for which the assumption of gene and environment independence in the source population, is often plausible [2, 3, 18, 31]. However, many of the variables used to subset injury case series for case-only analyses of exposure effect modification are socially patterned (e.g., alcohol use) or are associated with social patterning of behaviors (e.g., sex, age, race, mental health). Thus, it seems unlikely that the independence assumption will commonly hold in case-only studies of effect modification in injury risk. Moreover, establishing conditional independence in case-only data analyses by controlling for a variable(s) that explains the association between the two exposures is likely to be difficult to do in practice [18]. In our example analyses of FARS data, it is difficult to conceptualize all of the variables that might explain associations between age and alcohol consumption in the general population. Furthermore, like many hospital series or registries used in injury epidemiology, the FARS includes very limited data on the personal or behavioral characteristics of cases that can be used as covariates in a case-only analysis.

Thus, it is likely that the OR generated from many injury case-only studies are uninterpretable due to violations of the independence assumption, even after conditioning on available covariates. Among case-only studies we found that compare pedestrians injured or killed while under the influence of alcohol to pedestrians injured or killed while sober, none interpreted their results within an underlying cohort or case-control framework [22–28]. In addition, none of those studies placed their results within the context of estimating an exposure effect modification or noted the independence assumption [22–28]. These critiques apply more broadly to the use of these two case-only designs in injury epidemiology: these studies rarely state the hypothesis being tested by the design (i.e., exposure effect modification or etiological heterogeneity), nor do they relate the estimated case-only OR to the OR that would have been estimated in case-control analyses of the case-series under investigation. For example, Table 2 relates the designs of ten recent, purposively selected, case-only studies from the literature to the principles discussed here. Two can be classified as studies of etiological heterogeneity. The rest are studies of exposure effect modification; among most of these studies, the independence assumption is unlikely to hold.

## Conclusion

The case-only designs reviewed here are commonly used in injury epidemiology research, but in practice these analyses and their interpretation have not been rigorously connected to the epidemiologic study design literature. Discussions of the results of these two types of case-only studies in injury epidemiology rarely state the type of hypothesis being tested by the design (whether effect modification or etiologic heterogeneity) and rarely relate the estimated case-only OR to the OR that would have been estimated in case-control analyses if appropriate controls were available for the case-series under investigation. As such, we argue that these case-only studies in injury epidemiology are commonly misinterpreted, and the underlying assumptions are not stated in a way that supports critical assessment.

When the research goal is to understand distinct causal pathways relevant to injury prevention, case-only studies of etiological heterogeneity may have utility. However, results from such studies should be considered hypothesis-generating and further investigated in full cohort, case-crossover or case-control studies that can estimate causal effects. Conversely, because the independence assumption is unlikely to hold for many of the putative causes of injury, case-only studies of exposure effect modification are unlikely to be interpretable. When such studies are conducted, the researchers should (1) directly address the likely validity of the independence assumption; (2) conceptualize and describe causes of non-independence; and (3) establish conditional independence through inclusion of appropriate covariates in case-only regression analyses. Absent these steps, case-only designs that test for exposure effect modification are unlikely to be useful for understanding the etiology of injuries or for designing interventions.

## Supplementary Material

1866917_Sup_1

1866917_Sup_12

## Figures and Tables

**Table 1:** Results of analyses of the two different case-only designs applied to pedestrian fatalities in the 2017–2018 FARS data^1^

Case-only analysis of etiological heterogeneity for driver’s alcohol status: univariate and multivariate analyses								Case-only analysis of exposure effect modification for pedestrian age × pedestrian alcohol status		
	
	Driver’s alcohol status (Dependent variable) Univariate model			Driver’s alcohol status (Dependent variable) Multivariate model				Pedestrian’s alcohol status (Dependent variable)		
				
Pedestrian’s demographics	Positive (N)	Negative (N)	OR (95% CI)	Positive (N)	Negative (N)	OR (95% CI)	Pedestrian’s demographics	Positive (N)	Negative (N)	OR (95% CI)

Age										
16–20	47	516	1	47	516	1	16–20	106	275	1
21–29	157	1465	1.22 (0.85, 1.74)	157	1465	1.23 (0.86, 1.75)	21–29	559	575	2.75 (2.07, 3.66)
30–39	141	1722	0.89 (0.62, 1.28)	141	1722	0.90 (0.62, 1.29)	30–39	640	692	2.64 (1.98, 3.51)
40–49	179	1627	1.22 (0.85, 1.77)	179	1627	1.23 (0.85, 1.78)	40–49	635	652	2.70 (2.05, 3.59)
50–59	175	2204	0.90 (0.62, 1.31)	175	2204	0.90 (0.62, 1.31)	50–59	837	794	3.01 (2.32,3.89)
60+	180	3456	0.62 (0.44, 0.86)	180	3456	0.61 (0.44, 0.86)	60+	561	1663	0.97 (0.76, 1.31)
Sex										
Male				605	7723	1				
Female				274	3267	1.11 (0.95, 1.28)				
Race										
White				592	7379	1				
Black				174	2173	0.97 (0.80, 1.18)				
Native American				21	243	0.68 (0.40, 1.18)				
Asian				36	427	1.10 (0.75, 1.62)				
Other, Unknown				56	768	0.92 (0.66, 1.28)				

1. For pedestrian fatalities occurring within U.S. Metropolitan areas in 2017 or 2018.

**Table 2:** Interpretation of ten case-only studies from the literature

Stratification of the case series	Can the stratification variable be applied to a control series?	Type of case-only design	Risk factors tested	Independence assumption required?	Likelihood that independence assumption holds

Pedestrian injuries classified as fatal or not fatal [32]	No	Etiological heterogeneity	Pedestrian age and impairment with alcohol and/or drugs	No	NA
Car crashes with drivers categorized by injury severity [33]	No	Etiological heterogeneity	Driver age and sex, time of day, type of crash partner, intersection type, weather conditions and lighting conditions	No	NA
Fatally injured drivers categorized as positive of negative for cannabinoids [5]	Yes	Exposure effect modification	Urban/rural location, region of the US, state marijuana legalization, time of crash, and driver sex, age and race,	Yes	Unlikely
Fatally injured drivers classified as driving for work or non-work reasons [6]	Yes	Exposure effect modification	Vehicle type, vehicle age, driver sex, driver being alcohol or drug positive and holding a commercial driver’s license.	Yes	Unlikely
Car crashes where the drivers were classified as being under the influence of alcohol or not [13]	Yes	Exposure effect modification	Density per square mile of rideshare trips that were in progress at the time of the crash	Yes	Unlikely
Classified suicide decedents as being under the influence of alcohol [34]	Yes	Exposure effect modification	Sociodemographic characteristics of decedent, presence of psychiatric disorders, prior suicide attempts and recent adverse life events.	Yes	Unlikely
Intimate partner violence perpetrators classified as having mental illness or not at the time of the offense [35]	Yes	Exposure effect modification	Perpetrator age, sex, and employment status, and history of previous convictions, alcohol abuse and/or self-harm.	Yes	Unlikely
Categorized deaths as intimate partner violence related or not [11]	Yes	Exposure effect modification	Decedent’s age, race, year of death, and weapon used.	Yes	Unlikely
Adolescent suicide decedents classified as younger (10–14) and older (15–17) [36]	Yes	Exposure effect modification	Life stressors (e.g. intimate partner issue, physical health problem, mental health problem) and suicide risk factors (e.g. cutting, recent suicide of loved one).	Yes	Possibly
Suicide decedents classified physician versus non-physician [37]	Yes	Exposure effect modification	Decedent’s gender, marital status, age, mental health measures, experience of life stressors.	Yes	Possible for some predictors and unlikely for others
