# Supplementary figures and images for: Causal Inference with Case-Only Studies in Injury Epidemiology Research
Source: Curr Epidemiol Rep. Author manuscript; Available in PMC 2023 May 5. (PMC10161782; doi:10.1007/s40471-022-00306-8)

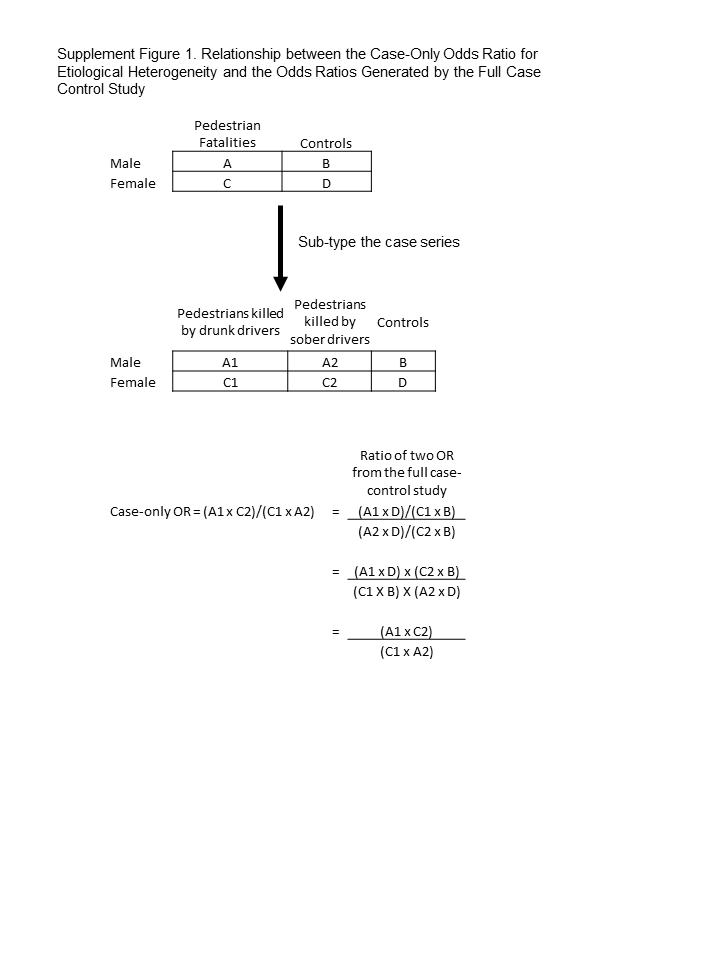

Supplement: 1866917_Sup_1 [file NIHMS1866917-supplement-1866917_Sup_1.tif]

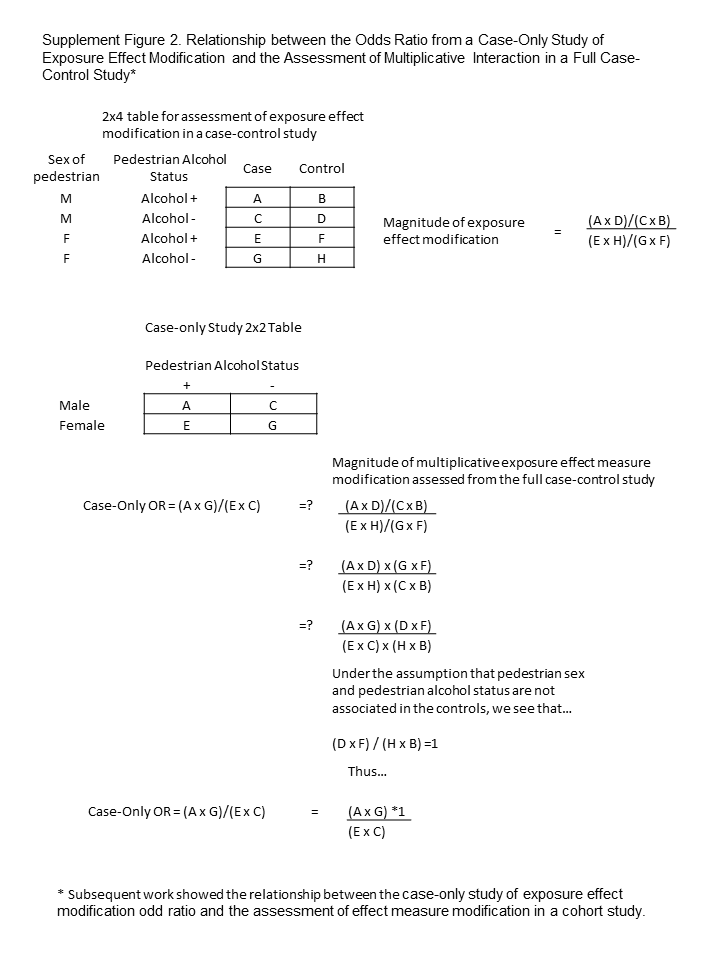

Supplement: 1866917_Sup_12 [file NIHMS1866917-supplement-1866917_Sup_12.tif]
